# Unexpected Ventilation Failure Following Intubation: Bronchospasm or Device Malfunction?

**DOI:** 10.7759/cureus.88850

**Published:** 2025-07-27

**Authors:** Ethan Steinberg, Gisele J Wakim, Rita El-Hachem, Manavii Kumar

**Affiliations:** 1 Anesthesia, University of Miami Miller School of Medicine, Jackson Memorial Hospital, Miami, USA; 2 Anesthesiology and Pain Medicine, American University of Beirut, Beirut, LBN

**Keywords:** ambulatory anesthesia, anesthesia complications, bronchospasm management, device recall, difficult airway algorithm, difficult airway management, difficult ventilation, endotracheal tube obstruction, endotracheal tube exchange, post-intubation complications

## Abstract

This case report describes the sudden intraoperative ventilation failure in an elderly patient undergoing thyroid lobectomy following intubation with a Medtronic neural integrity monitoring (NIM) endotracheal tube (ETT). Despite confirming appropriate tube positioning and initiating bronchodilator therapy, ventilation could not be restored until the NIM tube was removed and replaced with a standard ETT. This case predates a Class I FDA recall of Medtronic NIM tubes for design-related obstruction, raising the possibility that device malfunction may have contributed to the clinical presentation. This report highlights the importance of maintaining a broad differential diagnosis during airway emergencies and considering tube exchange early in the process.

## Introduction

Sudden failure to ventilate following endotracheal intubation represents a critical intraoperative emergency. The differential for post-intubation ventilation failure includes both mechanical obstruction (i.e., tube kinking, mucus plugging, tracheomalacia, or cuff overinflation) and physiologic causes such as bronchospasm [1]. Distinguishing between these entities in real time can be challenging, but prompt intervention is essential to avoid severe hypoxemia and hemodynamic collapse. Neural integrity monitoring (NIM) endotracheal tubes (ETT) are commonly used in thyroid surgery to monitor the recurrent laryngeal nerve intraoperatively [2]. However, these specialized tubes have a larger external diameter and can be more stimulating to the airway [3]. We report a case of an elderly patient without any known pulmonary comorbidities undergoing thyroid lobectomy who developed sudden ventilation failure following NIM tube placement, raising a challenging question of bronchospasm vs. tube obstruction and highlighting the importance of a structured response to airway emergencies.

## Case presentation

An 87-year-old ASA III female with a BMI of 19.5 presented for a left thyroid lobectomy for papillary thyroid carcinoma, incidentally diagnosed after a fall at home. Preoperative ultrasound demonstrated a 2x2 cm isoechoic nodule in the left lobe of the thyroid with a small cystic component. Her past medical history was significant only for a St. Jude Medical 2160 Endurity pacemaker placed six years prior for sick sinus syndrome. The device was interrogated as part of the preoperative workup, which was otherwise unremarkable. Complete blood count, electrolytes, TSH, and T4 were all within normal limits. The patient described some recent changes in her voice but denied any smoking history, palpable nodules in her neck, difficulty swallowing, or orthopnea. Airway exam revealed a Mallampati class II airway with normal thyromental distance and full neck range of motion.

General anesthesia was induced with lidocaine 2% (1.5 mg/kg), fentanyl (0.5 mcg/kg), etomidate (0.4 mg/kg), and rocuronium (0.4 mg/kg). After induction, the patient was able to be ventilated without any difficulty. Before intubation, the cuff was tested and was able to be inflated and deflated with no apparent issues. The trachea was intubated using videolaryngoscopy and a 7.0 NIM ETT with visual confirmation of the tube passing through the vocal cords. Immediately following cuff inflation, the lungs became difficult to ventilate with absent breath sounds bilaterally, no visible chest rise, and no end tidal CO_2_. The ETT was disconnected from the ventilator, and ventilation was attempted unsuccessfully with an Ambu bag. NIM ETT positioning was confirmed, and the tube was suctioned.

**Figure 1:**
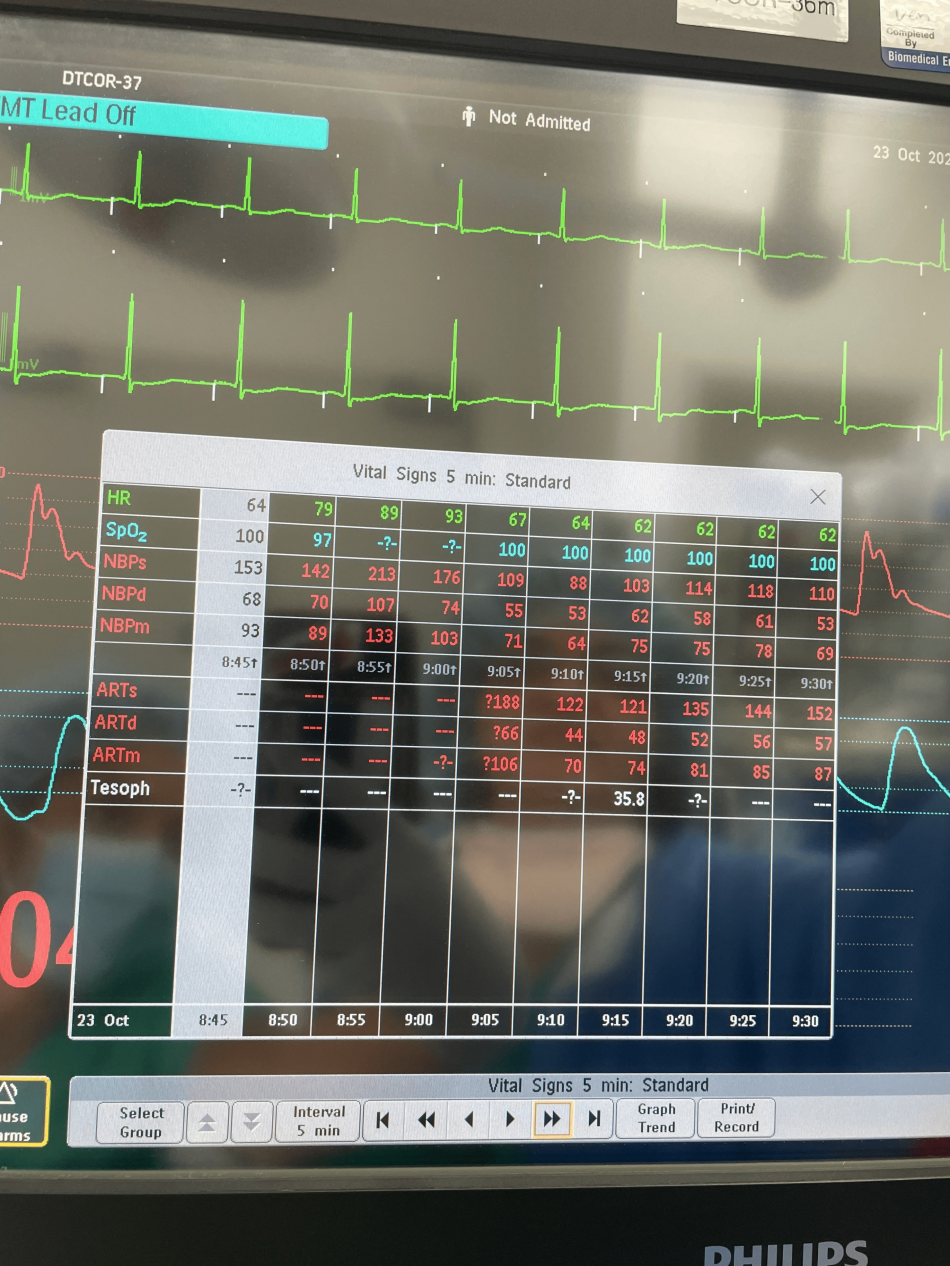
Vital signs throughout the course of treatment The NIM tube malfunction occurred between 8:55 and 9:00 am. During this period, the patient was hypertensive to 213/107. An arterial line was placed during the NIM tube malfunction. After replacement with a standard ETT, the patient’s blood pressure returned to baseline ETT: endotracheal tube; NIM: neural integrity monitoring

**Figure 2:**
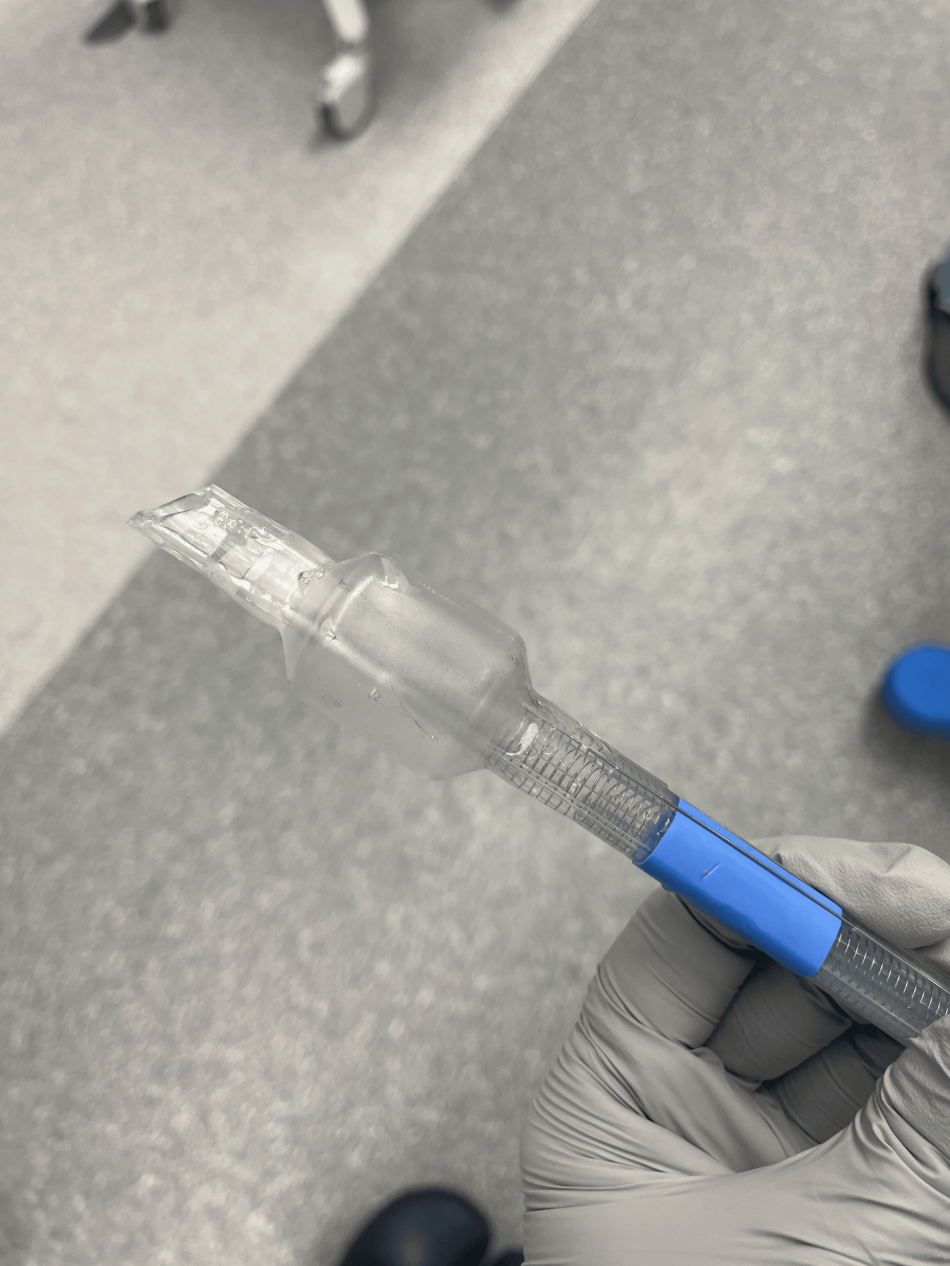
Inflating the used NIM tube to confirm the absence of air leaks NIM: neural integrity monitoring

**Figure 3:**
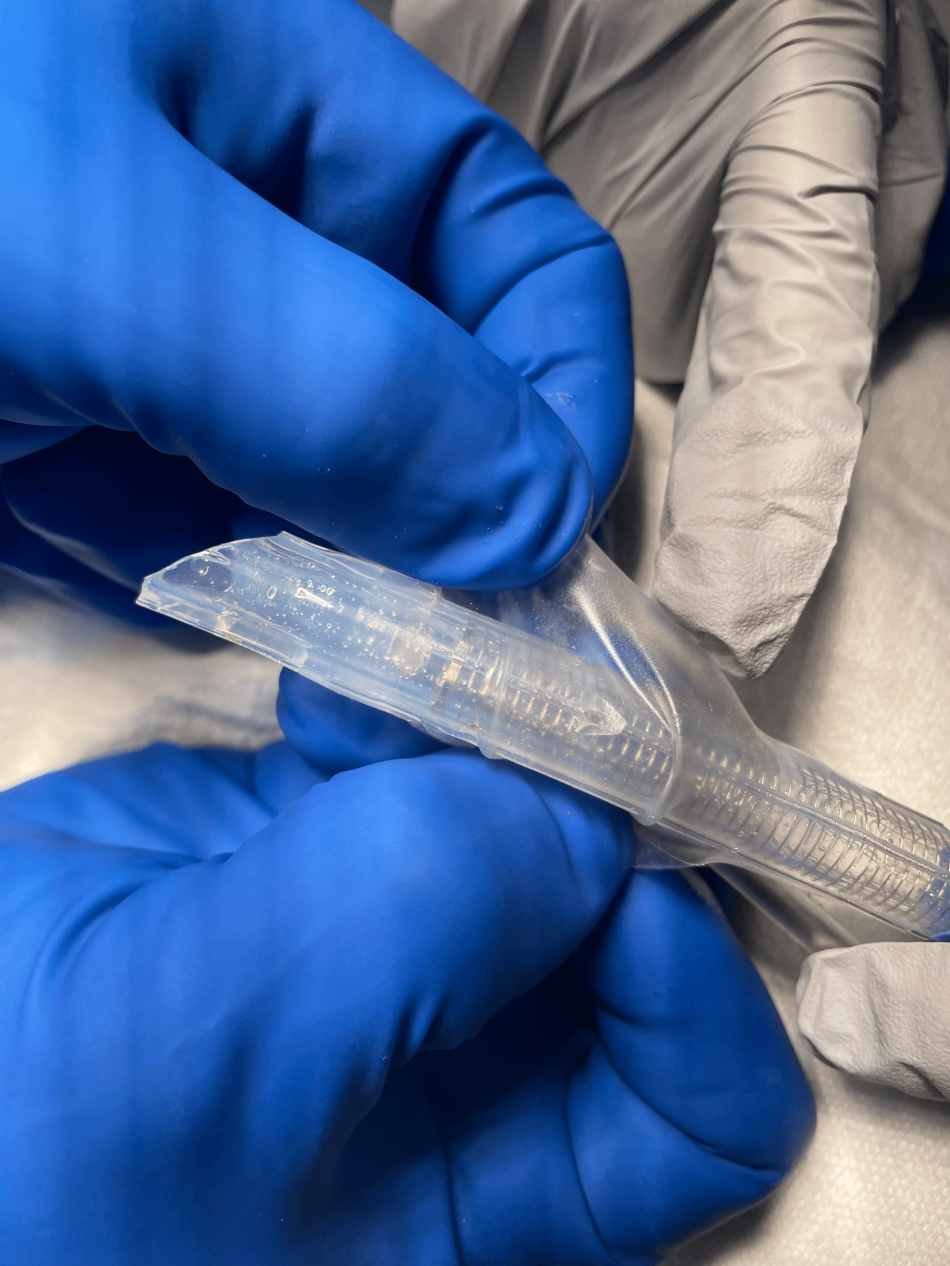
Close-up of used NIM tube confirming the absence of tears in the balloon cuff portion NIM: neural integrity monitoring

At this time, bronchospasm was suspected, and a total of 900 mcg of inhaled albuterol was administered, along with 20 mcg of IV epinephrine in 10 mcg increments. The patient remained hemodynamically stable with systolic blood pressures ranging from 150-165, heart rate 60-75, and SPO_2_ 100%. NIM ETT positioning was again confirmed using videolaryngoscopy. At this point, the patient desaturated to SPO_2_ of 72% and then lost pulse ox on the monitor; blood pressure increased to 213/107, peak airway pressure increased to 42 mmHg, and the anesthesia team called for additional assistance with airway management (Figure 1). The NIM ETT was removed, and the patient was re-intubated using a standard 7.0 ETT after which they were able to be successfully ventilated and SPO_2_ recovered to 100%. An additional 450 mcg of inhaled albuterol and 180 mcg of IV epinephrine were administered to maintain bronchodilation. During this period, an arterial line was placed for enhanced hemodynamic monitoring. After the patient was stabilized, the removed NIM tube was inspected and found to have no air leaks or visible obstructions (Figures 2, 3).

Due to this complication, the decision was made not to proceed with the surgery. A chest X-ray and 12-lead EKG were performed, which were both unremarkable, and an arterial blood gas showed appropriate oxygenation and a pH of 7.43. The patient emerged from anesthesia neurologically intact and without any complications and was transferred to recovery in stable condition. She was observed in the hospital overnight and discharged the next day.

## Discussion

This report highlights a rare but critical intraoperative complication: acute ventilation failure immediately following intubation with an NIM ETT in a patient without any known risk factors for reactive airway disease. Despite aggressive bronchodilator management, the event only resolved once the NIM tube was removed and replaced with a standard ETT. While bronchospasm under anesthesia is most commonly seen in patients with pre-existing asthma or COPD, it can also occur in patients without any pre-existing pulmonary disease [4,5]. Triggers include airway instrumentation during intubation and extubation, histamine release, excess vagal tone, and insufficient depth of anesthesia (volatile anesthetics like sevoflurane have bronchodilatory, anti-inflammatory, and vagal blocking properties) [6]. At the time of the case, bronchospasm was seen as the most likely diagnosis, especially after repeat video laryngoscopy confirmed proper placement of the tube and suctioning did not reveal any obstructive secretions.

Given the temporal relationship between the placement of the NIM ETT and the onset of symptoms, it is plausible that this device contributed to the patient’s presentation. NIM tubes are commonly used during thyroid surgery for intraoperative monitoring of the recurrent laryngeal nerve, but the electrodes they contain necessitate a larger outer diameter and increased rigidity compared to standard ETTs [2,3]. This can lead to greater mechanical stimulation of the airway during intubation or extubation, increasing the risk of reflex bronchoconstriction [4].

In July 2024, nine months after the event described in this report, the FDA issued a Class I recall for the Medtronic NIM ETTs due to the potential for the tube to become obstructed intraoperatively due to poor design [7]. This development raises the possibility that tube malfunction contributed significantly to the patient’s acute decompensation. Multiple previous case reports of sudden ventilation failure using NIM ETTs describe the potential for overinflation of the cuff to cause herniation and obstruction of the tube; however, this would not be visibly apparent after the cuff has been deflated and the tube removed [8,9]. Notably, breath sounds were absent bilaterally on auscultation. While this is possible in severe cases of bronchospasm that prevent any air movement at all, the more likely finding is expiratory wheezes. Additionally, bronchospasm should respond to albuterol and epinephrine, while this patient had no response to repeated doses of both medications. The fact that difficulty in ventilating resolved immediately upon removal of the NIM ETT and replacement with a regular ETT further supports the idea that the tube itself was a primary driver of this adverse event.

## Conclusions

This report illustrates the importance of maintaining a broad differential and a structured approach during intraoperative airway emergencies. Reconfirmation of tube placement, suctioning, a trial of bronchodilators, and ultimately tube exchange were all essential components of management. The decision to abort an elective procedure after this event prioritized patient safety. Anesthesiologists should remain vigilant when using specialized airway devices like NIM tubes, especially given the recent high-profile recalls. When ventilation fails without an obvious cause in a patient unresponsive to escalating bronchodilators, ETT tube failure should be high on the differential, even in the absence of visible obstruction.
